# Polyhydroxyalkanoates (PHAs): Biopolymers for Biofuel and Biorefineries

**DOI:** 10.3390/polym13020253

**Published:** 2021-01-13

**Authors:** Shahina Riaz, Kyong Yop Rhee, Soo Jin Park

**Affiliations:** 1 Department of Chemistry, Inha University, Incheon 22212, Korea; shahinariaz519@gmail.com; 2 Department of Mechanical Engineering (BK PLUS), College of Engineering, Kyung Hee University, Yongin 17104, Korea

**Keywords:** polyhydroxyalkanoates, biofuels, biorefineries, PHAs biofuels, renewable energy

## Abstract

Fossil fuels are energy recourses that fulfill most of the world’s energy requirements. However, their production and use cause severe health and environmental problems including global warming and pollution. Consequently, plant and animal-based fuels (also termed as biofuels), such as biogas, biodiesel, and many others, have been introduced as alternatives to fossil fuels. Despite the advantages of biofuels, such as being renewable, environmentally friendly, easy to source, and reducing the dependency on foreign oil, there are several drawbacks of using biofuels including high cost, and other factors discussed in the fuel vs. food debate. Therefore, it is imperative to produce novel biofuels while also developing suitable manufacturing processes that ease the aforementioned problems. Polyhydroxyalkanoates (PHAs) are structurally diverse microbial polyesters synthesized by numerous bacteria. Moreover, this structural diversity allows PHAs to readily undergo methyl esterification and to be used as biofuels, which further extends the application value of PHAs. PHA-based biofuels are similar to biodiesel except for having a high oxygen content and no nitrogen or sulfur. In this article, we review the microbial production of PHAs, biofuel production from PHAs, parameters affecting the production of fuel from PHAs, and PHAs biorefineries. In addition, future work on the production of biofuels from PHAs is also discussed.

## 1. Introduction

Polyhydroxyalkanoates (PHAs) are bacterial polyesters synthesized by several prokaryotic microorganisms under unbalanced nutrition conditions, e.g., carbon and energy storage conditions. Lemoigne first discovered these thermoplastics in 1926, and since then, PHAs have attracted significant commercial and research interest from the eco-friendly polymer market due to their favorable properties such as high biocompatibility, excellent biodegradability, diverse chemical structure, and manufacturing from renewable carbon resources [1,2,3,4,5].

Bio-based polyesters are considered alternates for petrochemical plastics. Moreover, among the various biopolymers, PHAs symbolize a sustainable future alternative for fossil fuel-based plastics. PHAs have mostly been synthesized by microbial cultures grown on renewable materials in clean environments [6,7,8,9]. Fossil fuels are energy recourses that fulfill most of the world’s energy requirements. However, their production and use cause severe health and environmental problems including global warming and pollution. Consequently, plant and animal-based fuels (also termed as biofuels), such as biogas, biodiesel, and many others, have been introduced as alternatives to fossil fuels. Despite the advantages of biofuels, such as being renewable, environmentally friendly, easy to source, and reducing the dependency on foreign oil, there are several drawbacks to using biofuels including high cost, and other factors discussed in the fuel vs. food debate. Therefore, it is imperative to produce novel biofuels while also developing suitable manufacturing processes that ease the aforementioned problems. Polyhydroxyalkanoates (PHAs) are structurally diverse microbial polyesters synthesized by numerous prokaryotic microorganisms. PHAs are naturally synthesized bio-based materials. Since PHAs are biocompatible, bioresorbable, and biodegradable; hence, they have no negative effect on the environment. When PHAs-based products are left in the environment, they degraded into CO_2_, H_2_O, and CH_4_, which facilitate the natural cycle of circulatory and renewability. In addition, the spent PHAs can undergo hydrolysis and produce optically pure building blocks which can be used as synthons for pharmaceuticals, organic synthesis, and fragrances [10]. Moreover, the structural diversity of PHAs allows them to readily undergo methyl esterification and to be used as biofuels, which further extends the application value of PHAs.

## 2. Sources of PHAs

**Figure 1:**
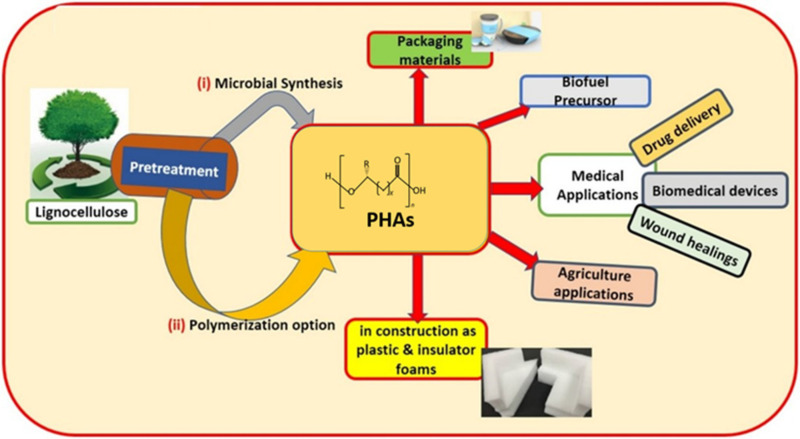
Applications of polyhydroxyalkanoates (PHAs) in various fields. Reproduced with permission from [17]. Copyright 2020 Elsevier Ltd.

PHAs are long polymeric molecules that exhibit ester linkages in their structures. PHAs are synthesized by various types of prokaryotic microorganisms as intracellular storage materials comprising significant amounts of carbon and energy [11]. A PHA molecule is typically made up of 600 to 35,000 fatty acid monomer units. Each monomer unit consists of a side chain (R) group that is usually a saturated alkyl group but can also be unsaturated, branched, or substituted alkyl group [12,13,14]. Depending on the number of carbon atoms in the PHA monomer, these biopolymers can be classified into short-chain length (scl) PHAs (generally 3–5 carbon atoms), medium-chain length (mcl) PHAs (comprising 6–14 carbon atoms), and long-chain length (lcl) PHAs (contains 15 or more carbon atoms). More than 150 PHA monomers have been recorded, where the chemical or physical modification of existing PHAs led to the production of new types of PHAs. These features of PHAs gave rise to a variety of PHA properties suitable for a number of applications, such as biodegradable packaging materials and various medical products, as shown in Figure 1 (Lignocellulose (biomass-derived) PHAs and their applications) [2,15,16].

**Figure 2:**
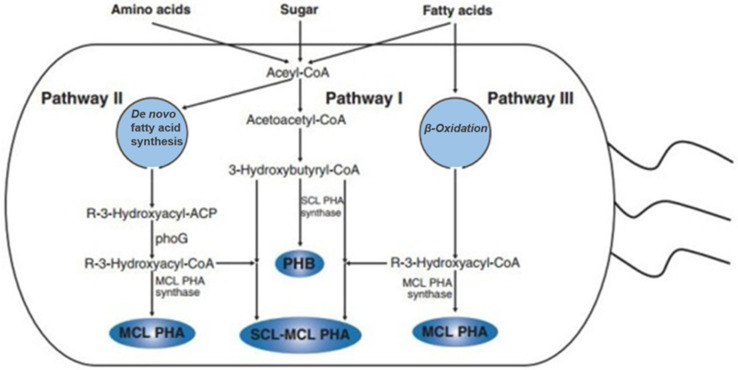
Biosynthesis of PHAs in bacteria. Reproduced with permission from [25]. Copyright 2015 Elsevier Ltd.

The synthesis of PHAs is a simple biosynthetic process consisting of only three genes and three enzymes. Figure 2 shows the PHAs biosynthetic pathway in bacteria. The Halobactericeae, a family of bacteria, contains more than 300 bacteria sources for PHA synthesis. *Pseudomonas oleovorans* and *Pseudomonas fragi* are bacteria that can produce mcl PHAs through the beta (β) oxidation of alkanoic acids by producing hydroxyalkanoyl-CoA as a substrate [17,18]. Furthermore, PHAs containing 2-hydroxypropionate (2HP), 3-hydroxypropionate (3HP), 4-hydroxybutyrate (4HB), 3-hydroxyvalerate (3HV), and mcl 3-hydroxyalkanoate (3-HA) monomer units can all be produced by *Escherichia coli* via genes or exogenous and endogenous processes [19,20,21]. In addition, the synthesis of PHAs was also studied in other eukaryotes, namely microalgae (both recombinant and even wild type). However, the bacterial synthesis of PHAs is not cost-effective compared to petroleum-based plastics; therefore; there is great interest in discovering a synthetic pathway for PHA production in eukaryotic cells, especially in crops [22,23,24].

### 2.1. Production of PHAs

**Figure 3:**
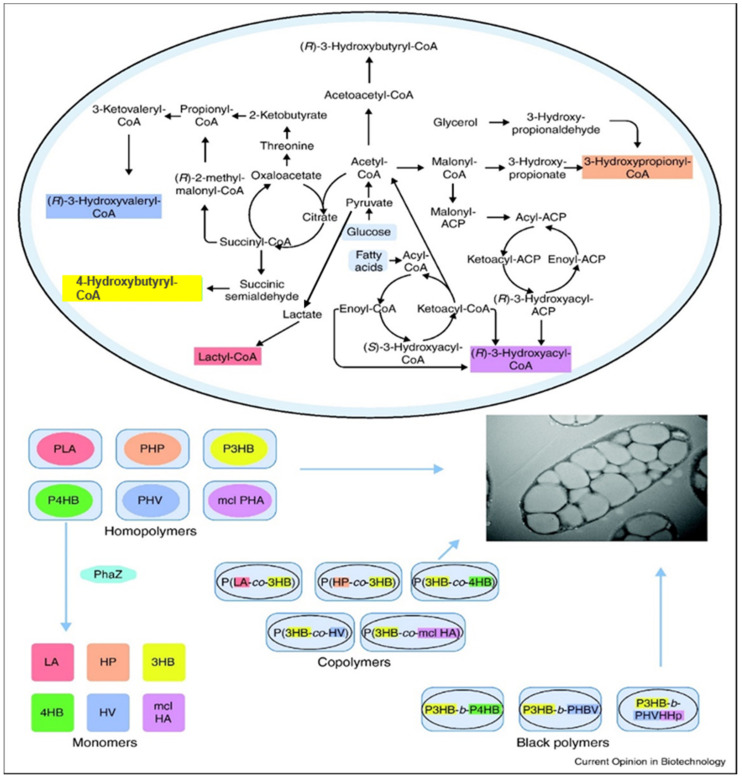
Microbial synthesis of PHAs. Key metabolic paths for the production of precursors for PHAs Reproduced with permission from [30]. Copyright 2011 Elsevier Ltd.

In the 1980s, numerous companies tried to produce PHAs on an industrial scale based on the expectations that petroleum prices would increase over time. People would prefer to use eco-friendly biodegradable plastics or green plastics instead. Moreover, industries such as Chemie Linz AG, Austria; ICI, UK; and TianAn, China, successfully produced poly-(*R*)-3-hydroxybutyrate (P3HB) and several copolymers such as P3HBHHx of (*R*)-3-hydroxybutyrate (3HB), (*R*)-3-hydroxyhexanoate (3HHx), P3HBV of (*R*)-3-hydroxybutyrate (3HB), and (*R*)-3-hydroxyvalerate (3HV) [1]. However, up until the year 2000, petroleum prices did not increase as significantly as expected and many PHA production-related projects were discontinued. Surprisingly, after 2001 a sharp increase in the petroleum price was observed and in 2008, a barrel of oil reached $140, which reignited the search for petroleum-free plastics (Figure 3 shows the production of PHAs). Industry focused on two polyesters: polylactic acid (PLA) and PHAs, where PLA is economical and available in bulk but PHAs are expensive. However, the applications and properties of PHAs can be tailored by varying the co-monomer and the content of the copolyesters [26,27,28,29]. The advantages and disadvantages of both PLA and PHAs are compiled in Table 1.

### 2.2. Fermentation Industry

Both wild type and recombinant bacteria are used to produce PHA (Table 2). The production of PHAs at an industrial scale has several prerequisites such as strain development, shake flask optimization, lab and pilot fermenter studies, and industrial production scale-up [31]. Furthermore, several factors affect the microbial production of PHAs including growth rate, cell density, percentage of PHAs in cell dry weight, the time required to attain final cell density, choice of substrate, price of the substrate, and the use of an economical method for PHAs extraction and purification [32,33,34,35].

### 2.3. Microbial Synthesis of PHAs Homopolymers

A homopolymer can be defined as a polymer that comprises >99% by weight of one type of monomer and <1% by weight of another. PHAs can be produced as homopolymers, copolymers, or blends depending on the bacterial strain or growth substrate used, and more than 300 species, mainly of bacteria, are reported to produce these polymers [36]. The bacteria that produce PHAs are categorized into two groups based on the stressed conditions required for PHA synthesis. One group requires the limitation of essential nutrients such as nitrogen (N), sulfur (S), magnesium (Mg), or phosphorus (P) with excessive carbon source supply. *A. eutrophus*, *Protomonas extorquens,* and *P. oleovorans* are included in this group. On the other hand, the second group does not require the limitation of essential nutrients bacteria (commonly known as growth-associated PHA biosynthesis). *Alcaligenes latus*, a mutant strain of *Azotobacter vinelandii* and recombinant *E. coli* belongs to the second group of bacteria [37]. P3HB, the most common homopolymer of PHA, is synthesized by wild type bacteria. Other homopolymers includes P3HP [31,38], P4HP [39], poly(3-hydroxyvalerate) (PHV), [40] poly(3-hydroxy-5-phenylvaleric acid) (P3H5PV) [41] poly(3-hydroxyhexanoate) (PHHx) [42], poly(3-hydroxyheptanoate) (PHHp) [43], poly(3-hydroxyoctanoate) (PHO) [44], poly(3-hydroxynonanoate) (PHN [45]), poly(3-hydroxydodecanoate) (PHD) [46], and poly(3-hydroxydodecanoate) (PHDD) [30,47]. These homopolymers have not yet been fully investigated due to their low contents in cells. The mcl-PHA homopolyesters are typically produced only by recombinant strains, e.g., β-oxidation weakened mutants [48].

*Cupriavidus necator* and recombinant *E. coli* are the most used strains for the industrial production of PHAs. Pohlmann et al., discovered the production of PHA homopolymer and copolyers, including P3HB (over 80%), P3HB4HB (over 75%), and P3HBV (over 75%), by *C. necator* and *E. coli.* Moreover, these results led to further genetic manipulations of bacteria for the production of PHAs. *A. latus* is another strain (similar to *R. eutropha*) that was also used for the production of P3HB and P3HBV [49,50,51].

**Figure 4:**
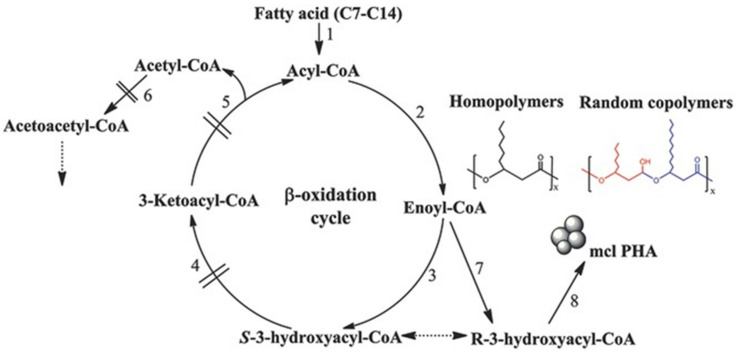
Production of mcl-PHA homopolymers by the β-oxidation of impaired *Pseudomonas entomophila* LAC23. Reproduced with permission from [55]. Copyright 2017 John Wiley & Sons, Inc.

Wang et al., [52] and Liu et al., [53] used *Pseudomonas putida* KT2442, a well-studied producer of PHA, to synthesize PHA homopolymers including PHHx and PHHp. Moreover, they synthesized an almost homopolymer of poly(3-hydroxyoctanoate-*co*-2mol% 3-hydroxyhexanoate) (PHO*) when hexanoate, heptanoate, and octanoate were used in the growth medium. The *pha*G gene of *P. putida* KT2442, encoding for *R*-3-hydroxyacyl-ACP-CoA transacylase and β-oxidation related genes, had been deleted preventing fatty acid substrates’ shortening. This leads to the accumulation of mutant to produce mcl PHA homopolymers. On the other hand, when PHA synthase genes *pha*C1 and *pha*C2 were exchanged with PHA synthase operon *pha*PCJ from *Aeromonas hydrophila* 4AK4, or *P3HBC* from *R. eutropha*, PHV or P3HB were produced using valerate, butyrate, and γ-butyrolacton as substrates, respectively. In addition, Liu and Chan [54] successfully synthesized poly(3-hydroxytetradecanoate) (PHTD) using *fadB* and *fadA* knockout mutant of *P. putida* KT2442. In another study, Wang et al., [55] used an engineered strain of *Pseudomonas entomophila LAC23* (designed from *P. entomophila* L48) and deficient β-oxidation pathways to produce mcl PHA homopolymers. The authors found that *P. entomophila LAC23* could be used for the synthesis of different mcl PHAs when using appropriate fatty acids as carbon sources (Figure 4) 3-hydroxytetradecanoates (3HTD) was successfully synthesized when tetradecanoic acid was used as carbon source. Moreover, the authors were able to produce C7-C14 PHA homopolymers using *P. entomophila* LAC23 grown on appropriate C7-C14 fatty acids.

Sharma et al., [56] used recombinant *P. putida* LS461, with the *phaC1pha ZphaC2* genes deleted, developed by involving JC123 carrying *phaC1_16_* PHA synthase. The resulting strain, *P. putida* LS46123, was able to produce P3HB or PHV PHA homopolymers when either glucose or free fatty acids were used as a carbon source. Wu et al., [57] demonstrated the production of P3HB *Rhodopseudomonas palustris* WP3-5 when acetate and propionate were used as carbon sources. Scheel et al., [58] developed recombinant *E. coli* strains by deleting *arcA* and *ompR;* two global regulators capable of preventing the uptake and activation of exogenous fatty acids. It was found that the yields of homopolymers P3HB, PHV, and PHHx increased significantly with only a modest increase in PHHp and PHD in Δ*arcA* mutant compared to the parental strain. Thus, *P. putida* KT2442, *P. putida* LS461, *P. entomophila LAC23*, *R. palustris* WP3-5, and *E. coli*-based derivatives can produce PHAs homopolymers. Table 3 summarizes the production of PHA homopolymers by different strains.

### 2.4. Microbial Synthesis of PHAs Copolymers

When two or more different types of monomers are linked in the same polymer chain, copolymers are formed [84]. All the mcl PHAs are copolymers of C6–C12. P3HB is the most commonly used PHA; due to its exceptional properties, such as high thermal stability and excellent mechanical properties, P3HB based copolymers have attracted industrial interest [6]. Various copolymers such as P3HBV, P3HB4HB, and P3HBHHx have been produced on an industrial scale for several applications, but the non-3-hydroxybutyrate (3HB) monomer of these copolymers is not economical and adds additional cost to the overall synthesis. For example, to produce copolymers of 3-hydroxyvalerate (3HV), 4-hyroxybutyrate (4HB), and 3-hydroxyhexanoate (3HHx) based on 3HB, propionate, 1,4-butandiol, and lauric acid are required. Moreover, these non-3HB monomers are toxic and challenging to control in the cell growth processes. As a consequence, researchers started using genetically engineered metabolic pathways and low-cost non-fatty acid substrates, such as glycerol, gluconate, and glucose, for the production of PHA copolymers [48,85].

**Figure 5:**
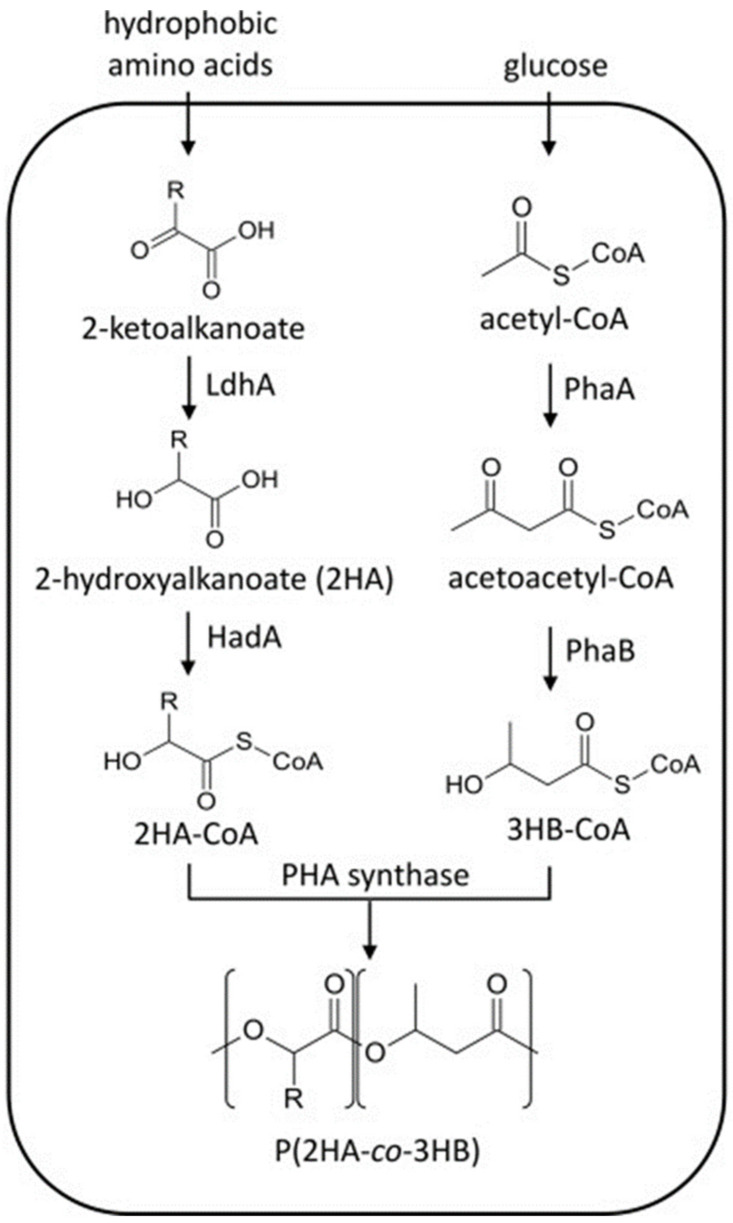
Poly (2-hydroxyalkanoate-co-3-hydroxybutyrate) (P2HA-co-P3HB) biosynthesis pathways in engineered *Escherichia coli*. Reproduced with permission from [113]. Copyright 2020 Elsevier Ltd.

Koller et al. [86], reported the synthesis of PHBV from 3HV-unrelated resources glucose and glycerol without the genetic manipulation of *Haloferax mediterranei* strain. Aldor et al., [87] successfully synthesized P3HBV by adopting a metabolically engineered pathway. The authors used a strain of *Salmonella enterica* serovar Typhimurium to metabolize propionyl coenzyme A (propionyl-CoA). The *S. enterica* accumulated significant amounts of P3HBV when grown aerobically using glycerol as a glucose source. Li et al., [85] constructed a metabolically engineered *E. coli* strain for the synthesis of P3HB4HB using glucose as a carbon source. The genes responsible for succinate degradation and accumulation of P3HB in *Clostridium kluyveri* and *R. eutropha*, respectively, were co-expressed for the synthesis of P3HB. Additionally, the *sad* and *gab*D genes of *E. coli* were deleted for the synthesis of the 4HB monomer. In another study, Zheng et al., [88] genetically engineered *A. hydrophila* 4AK4 and *P. putida* GPp104 for the production of P3HBHHx copolymers using gluconate and glucose as carbon sources. A truncated gene (*tesA*) encoding for cytosolic thioesterase I of *E. coli*, which is used for the conversion of acyl-ACP into free fatty acids, was incorporated into *A. hydrophila* 4AK4. The recombinant strain was able to synthesize 10% and 19% (*w*/*w*) P3HBHHx when grown on gluconate and glucose, respectively. Table 4 summarizes the production of PHA copolymers by different recombinant strains. Sudo et al., reported the synthesis of (2-hydroxyalkanoate-*co*-3-hydroxybutyrate) (P2HA-*co*-P3HB) in engineered *E. coli* using glucose as a carbon source and hydrophobic amino acids as supplementation. Four different amino acids, i.e., leucine, valine (Val), isoleucine (Ile), and phenylalanine, were introduced into the growth medium and P2HB-*co*-P3HB production was observed when Val was used as supplementation. Figure 5 shows the biosynthetic pathway of P2HB-*co*-P3HB in *E. coli* from biomass-derived carbon sources.

### 2.5. Microbial Production of PHA Block Copolymers

**Figure 6:**
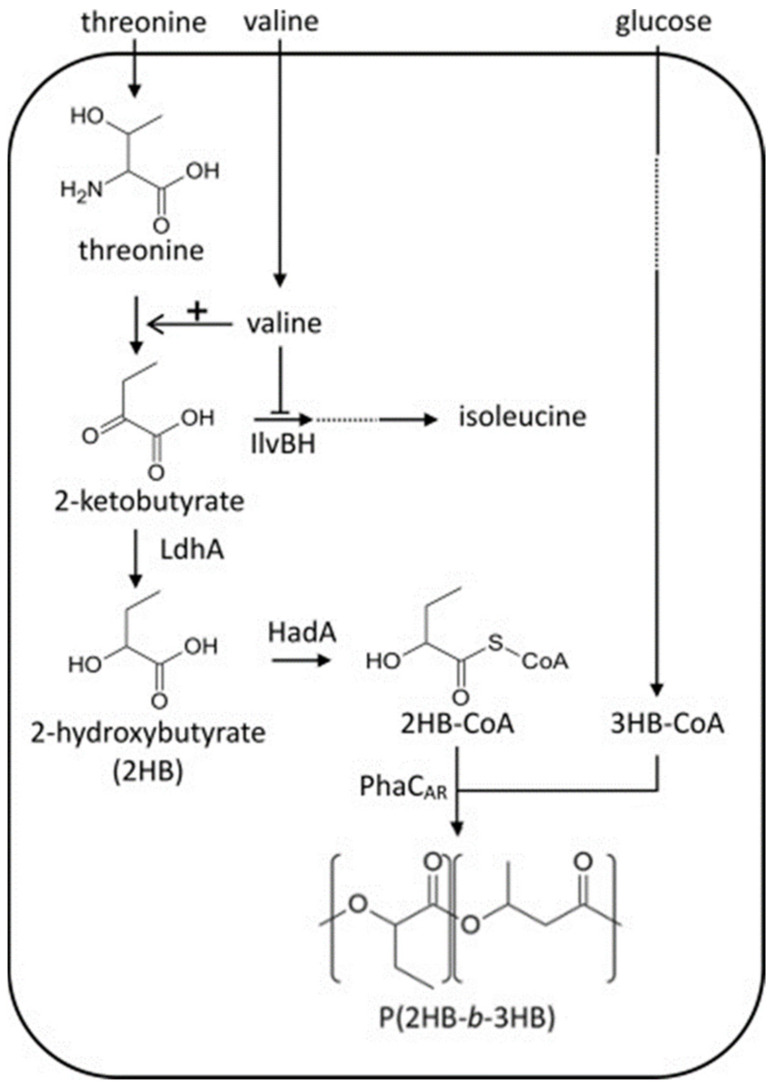
Poly(2-hydroxybutyrate-b-3-hydroxybutyrate) (P2HB-b-P3HB) biosynthesis pathways in engineered E. coli with amino acid supplementation. Amino acids are also supplied through the endogenous pathways. Reproduced with permission from [113]. Copyright 2020 Elsevier Ltd.

A polymer that contains alternating segments of different polymer compositions, covalently linked via their reactive ends, is called block copolymers. Block copolymers can be in the form of diblock, triblock or repeating multiblocks. Studies have shown that PHA homopolymers and copolymers exhibit many unfavorable properties and a common setback in the commercialization of these polymers is their brittleness, which limits their end-use applications [114]. Therefore, significant research focus has been placed on developing a suitable combination of these PHA polymers to achieve desirable properties [115]. Pederson et al., [116] and McChalicher et al., [115] successfully synthesized P3HB-*b*-P3HBV block copolymers in *C. necator* with the periodic addition of substrates (fructose and pentanoic acid). The P3HB and P3HBV blocks were synthesized during the fructose utilization and pulse feed of pentanoic acid. Though, the P3HB-*b*-P3HBV block copolymers still exhibited brittleness due to the brittle nature of scl PHAs. However, the combination of scl and mcl block copolymers could result in better properties compared to pure scl or mcl PHAs copolymers. In this context, Li et al., [117] reported the synthesis of P3HB-*b*-PHVHHp (P3HB-*b*-poly(3-hydroxyvalerate-co-3-hydroxyheptanoate) (PHVHHp)) block copolymers by recombinant *P. putida* KTOY06ΔC *(phaPCJ _A.c_ )*. The authors demonstrated the formation of block copolymers when butyrate (C4) and heptanoate (C7) were introduced into the growth media. Keeping this in view, Tripathi et al., [118] adopted the same strategy and microbially linked scl-P3HB and mcl-HHx to synthesize P3HB-*b*-PHHx diblock copolymers in a β-oxidation deficient recombinant *P. putida* KT2442. The β-oxidation cycle was deleted to its maximum and glycerol was used as a carbon source. Recently, Sudo et al., [113] were able to synthesize poly(2-hydroxybutyrate) (P2HB)-*b*-P3HB polymers in *E. coli* using glucose as carbon source and hydrophobic amino acids as supplementation. The authors demonstrated that block copolymers successfully accumulated in genetically engineered *E. coli* by expressing the chimeric PHA synthase (PhaC_AR_) gene. It was demonstrated that PhaC_AR_ exhibits strict substrate specificity and that it can synthesize P2HB-*b*-P3HB in engineered *E. coli* from exogenous 2HB and 3HB. Moreover, 2-hydroxyalkanoate (2HA) units were incorporated using PhaC_AR_, the lactate dehydrogenase (LdhA), and CoA transferase (HadA). Four hydrophobic amino acids, leucine, Val, Ile, and phenylalanine, were introduced into the culture as supplementation and the required block copolymer was produced. Figure 6 shows the biosynthetic pathway of P2HB-*b*-P3HB in *E. coli* from biomass-derived carbon sources.

## 3. Biofuels Based on PHAs

Due to the scarceness of traditional fossil fuels, increased emissions of combustion pollutants, and their costs, biomass-based fuels have become an attractive alternative option. Biofuels refer to the fuels produced from organic materials such as plants and animals. Table 5 lists the types of biofuels used for energy generation [119]. Studies have shown that the depletion of fossil fuels and environmental concerns increased the interest among scientists to explore renewable biofuels that are environmentally friendly and more acceptable. More importantly, in recent years the worldwide interest in biofuels has increased enormously [120]. To date, several biofuels derived from biomass have been explored including hydrogen (H_2_), methane (CH_4_), methanol (CH_3_OH), ethanol (C_2_H_5_OH), biodiesel, acetone (C_3_H_6_O), and several others. Globally, the transportation industry is a major source of carbon dioxide (CO_2_) emission and energy consumption. Liquid biofuels can replace fossil fuels in the transportation industry [121]. Liquid biofuels, such as biodiesel and bioethanol, have numerous advantages including ease of production from common biomass, eco-friendly, biodegradable, and sustainability. However, the extensive use of these biofuels created the food vs. fuel controversy, while their high production costs restricted their large-scale application in the transportation industry.

**Figure 7:**
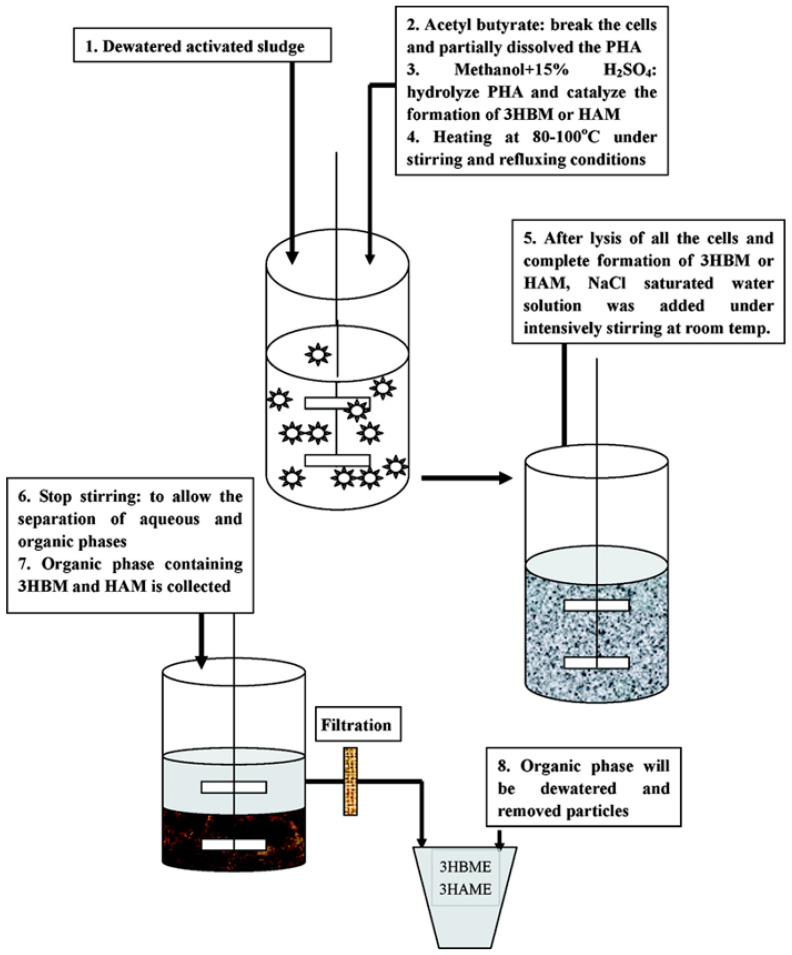
Production of PHA based biofuels hydroxyalkanoates methyl ester (HAME) or hydroxybutyrate methyl ester (HBME). Reproduced with permission from [122]. Copyright 2009 American Chemical Society.

In 2009, Zhang et al., [122] introduced a novel biofuel, hydroxyalkanoates methyl ester (HAME) and hydroxybutyrate methyl ester (HBME), derived from bacterial PHAs (Figure 7). HA esters have chemical structures similar to those of biofuels, especially biodiesel, consisting of the methyl ester of long-chain fatty acids. HA esters are different from petroleum: petroleum is enriched with low oxygen (O_2_) and high nitrogen (N_2_) and sulfur (S) contents leading to the generation of environmental pollutants upon combustion. In contrast, HA esters have a high O_2_ content without any N_2_ or S. In general, oxygenated additives can reduce the exhaust smoke for diesel as well as decrease combustion times and ignition delay. However, oxygenated additives, like HA esters, have a low cetane number and a high heat of vaporization which makes it difficult to completely fuel diesel engines. Therefore, the introduction of HA esters as sustainable fuels or fuel additives contributed to the divergence of the biofuel market [122]. In contrast, the use of PHAs as biofuel sources becomes very promising, as highly purified PHAs are not needed. Thus, PHAs can be produced from the sewage or industrial wastewater, which is not in competition with human or animal food sources, leading to cost reduction.

**Figure 8:**
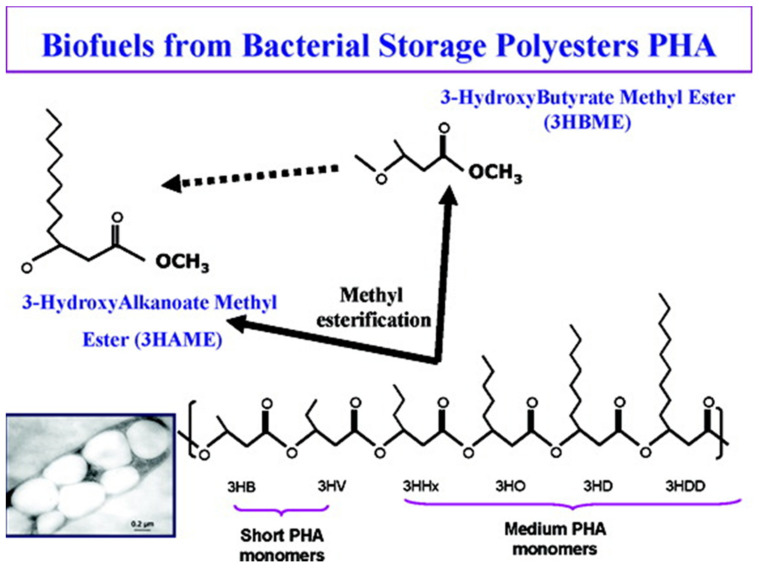
Methyl esterification of various PHA monomers to produce PHA-based biofuels. Reproduced with permission from [122]. Copyright 2009 American Chemical Society.

Zhang et al., [122] reported the production of HAME or HBME via the esterification of mcl PHAs and P3HB by acid-catalyzed hydrolysis (Figure 8). Combustion heat is one of the most important benchmarks to assess the quality of a fuel. The combustion heat of HAME, HBME, and their blends with diesel, gasoline, and C_2_H_5_OH were compared. The results demonstrated that when HBME was blended with C_2_H_5_OH, the combustion heat of C_2_H_5_OH improved by up to 30%. However, the combustion heat was reduced when HBME was blended with gasoline and diesel. Furthermore, HBME and HAME were obtained via acid-catalyzed hydrolysis and the most commonly used acid catalyst is sulfuric acid (H_2_SO_4_). Moreover, until now, different concentrations of H_2_SO_4_ with methanol were used to reach the maximum yield of HBME or HAME.

Choonut et al., [123] documented the optimized conditions for HBME production from P3HB by acid-catalyzed hydrolysis. They produce P3HB from *A. eutrophus* TISTR 1107 and used it as a substrate to produce HBME using three acids (H_2_SO_4,_ HCl, and H_3_PO_4_) at three different concentrations (5, 10, and 15%) and with different types of solvents including methanol and ethanol. The production of HBME was performed at various reaction times of 10, 20, 30, 40, 50, 60, and 70 h. Moreover, the optimal conditions to produce HBME were methanol with 10% (*v*/*v*) H_2_SO_4_ at 67 °C for 50 h. The maximum yield of HBME (70.7%) was obtained under these specific conditions. Sangkharak et al., [124] reported the production of HBME from P3HB. P3HB was isolated from *Bacillus licheniformis* M2-12 and used as a biofuel substrate. P3HB underwent esterification under acid- and base-catalyzed reaction conditions. The acid-catalyzed reaction proceeded using methanol with 10% (*v*/*v*) H_2_SO_4_ at 67 °C for 60 h. In contrast, the base-catalyzed hydrolysis of P3HB was performed using methanol with 2% (*w*/*w*) potassium hydroxide at 67 °C for 60 h and exhibited a high recovery of 68%. The fuel-related properties and purity (>95%) of the HBME produced from the acid-catalyzed hydrolysis of P3HB were investigated. The high molar mass, carbon and oxygen content, freezing point, and density of HBME showed that it can be potentially used as novel biofuel or as a fuel additive. The increasing need for biofuels may also increase the demand for biofuel additives or lubricants. Keunun et al., [125] reported the accumulation of P3HB in *C. necator*, by a two-step fermentation process that utilizes this P3HB as a novel substrate, for the synthesis of biolubricants. The lubricant was produced via a two-step transesterification process.

## 4. Parameters Affecting HAME-Based Biofuels Production

Several factors that affect the yield of HAME biofuels have been identified. These include reaction time, temperature, type and content of alcohol, as well as catalyst type and concentration.

### 4.1. Reaction Time

The conversion of fatty acids to esters is directly proportional to the reaction time. At the start, the dispersion of alcohols into the PHAs slows down the reaction rate, however, the reaction rate increases after thorough mixing. In general, the product yield reaches a maximum in < 90 min, after which it remains relatively constant upon a further increase in reaction time. Choonut et al., [123] studied the effect of reaction time (i.e., 10, 20, 30, 40, 50, 60, and 70 h) on the conversion of P3HB into HBME. Results showed that while the yield of HBME increased from 12.8 to 70.7% with increasing reaction time from 10 h to 50 h, the yield decreased upon a further increase in reaction time (60 and 70 h). Hence, a long reaction time is needed to produce HBME from P3HB under optimum reaction conditions. Okwundu et al., [126] reported the production of biodiesel from low free fatty acid beef tallow using homogenous and heterogeneous base-catalyzed reactions while documenting the effect of reaction time on the yield. The homogeneous and heterogeneous catalysts give a maximum biodiesel yield of 94.2% and 87.5% at 1 h and 4 h reaction time, respectively.

### 4.2. Reaction Temperature

Studies have shown that a high reaction temperature decreases the viscosity of fatty acids, which leads to an increase in reaction rate and a reduced reaction time. However, increasing the reaction temperature above a certain level results in a decrease in yield due to the saponification of triglycerides. In general, the reaction temperature must be lower than the boiling point of alcohol used. Base-catalyzed alcoholysis is normally performed near the boiling point of the alcohol or even at room temperature [74]. Zhang et al., [69] obtained a maximum yield (52%) of HBME from P3HB at 100 °C. In contrast, Wang et al., [75], Keunun et al., [72], Sangkharak et al., [71], and Choonut et al., [70] perform the esterification reaction of P3HB at 67 °C and obtained maximum yields for HBME of 40, 65, 68, and 70%, respectively. Overall, the optimal temperature ranges for the esterification of P3HB ranges from 67 to 100 °C, depending on other reaction parameters.

### 4.3. Type and Content of Alcohol

The alcohols used in the esterification reactions include CH_3_OH, C_2_H_5_OH, propanol (C_3_H_7_OH), and butanol (C_4_H_9_OH), of which CH_3_OH and C_2_H_5_OH are the most used. These two alcohols are the first choice for laboratory-scale research owing to their low cost and physical and chemical properties. In general, a higher amount of alcohol leads to a higher esterification conversion of the polyesters to biofuels. Weerachanchai et al., [127] perform the acid-catalyzed esterification of palm shell oil and investigated the effect of alcohol type and content on the esterification conversion. The results demonstrated that a 3.25:1 mole ratio of CH_3_OH or C_2_H_5_OH to acid under optimum conditions led to a 73.39% and 54.80% esterification conversion, respectively. However, upon a twofold increase in the mole ratio of alcohol, the conversion only increased by 2.42%. CH_3_OH gave higher esterification conversion compared to C_2_H_5_OH, which could be ascribed to the length of the alkyl group (C1 vs. C2) where the higher solubility and activity of the scl alcohol (CH_3_OH) promotes a faster reaction rate. Long-chain alcohols result in longer reaction times at the same reaction temperature of the scl alcohols, or shorter reaction time at higher reaction temperatures [128]. Choonut et al., [123] studied the effect of the alcohol used (CH_3_OH and C_2_H_5_OH) on the esterification of P3HB to produce HBME and found that CH_3_OH with 10% acid catalyst gives the highest yield.

### 4.4. Catalyst Type and Concentration

In general, acid- and base-catalyzed systems are used for the esterification of polyesters. Acid-catalyzed systems are mostly used to produce HAME biofuels, for which H2SO4 is the most frequently used catalyst. However, other acids such as H_3_PO_4_, organic sulfonic acids, and HCl are also used. Zhang et al., [69], Wang et al., [75], Keunun et al., [72], and Sangkharak et al., [71] investigated the esterification of PHAs to produce HAME using H2SO4 as catalyst. Choonut et al., studied the effect of different types of acids, including H_3_PO_4_, H_2_SO_4_, and HCl, on the conversion of P3HB to HBME. Their findings suggested that an acid-catalyzed system containing H_2_SO_4_ as catalyst gives maximum yield.

Catalyst concentration affects the yield of HAME biofuels. In general, the product yield increases when a 10% (*v*/*v*) acid concentration is used, but decrease upon a further increase in catalyst concentration. Zhang et al., [69], studied PHAs conversion to HAME using 15% H_2_SO_4_ and reported a 52% conversion. In contrast, Wang et al., [75], Keunun et al., [72], Sangkharak et al., [71], and Choonut et al., [70] carried the conversion of P3HB to HBME and reported maximum yield at this concentration.

## 5. PHAs Biorefineries

**Figure 9:**
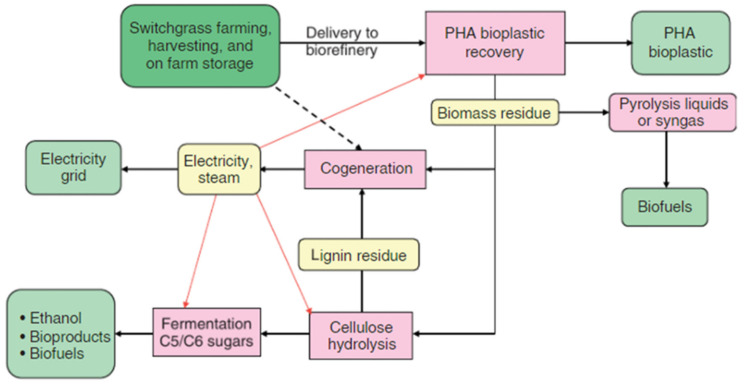
Biorefineries for the production of PHAs from switchgrass. Reproduced with permission from [131]. Copyright 2009 Society of Chemical Industry and John Wiley & Sons, Ltd.

The United Nations identified numerous crops worldwide that can be used as bioenergy feedstocks. Many of these crops have the potential for coproducing PHAs. In the crop-based synthesis approach of PHAs, a metabolically engineered system is established in one crop and, at the same time, generic technology is created which can be transferred to a different biomass crop. In this way, additional crops will be harvested and commercialized in different regions based on need [129,130]. The sugarcane industry was the first PHAs producing biorefinery. This industry faced many challenges, which were clearly relevant to dedicated bioenergy crops where the residual bagasse is combusted to produce steam and power for the process while excess power is returned to the power grid [80]. The major advantage of sugarcane is that 42% of the total dry weight of the stalk can be extracted to fermentable sugar. However, another bioenergy crop, switchgrass, holds potential for multigene expression systems to coproduce PHAs. Moreover, switchgrass holds the advantage over sugarcane as it can be grown in less moist environments and can be processed year-round, leading to overall improved capital efficiency. Moreover, switchgrass is a high yield crop, meaning it can be harvested on land set aside for marginal use of other crops, needs few chemical supplies, can potentially reduce the runoff of topsoil, pesticide, and fertilizers, and fixes CO_2_ in its roots systems [81] (Figure 9).

In summary, the solvent extraction process and residual dry biomass of switchgrass are used to recover PHAs and to produce bioenergy in cogeneration (cogeneration offers efficient generation of both steam and electricity while the excess steam can be used in other biorefinery processes) [82]. Unlike fully integrated switchgrass-based PHAs biorefinery, PHAs biorefinery can be as simple as the installation of a recovery system near an existing power plant which provides a reliable economical supply of dry and easily handled feedstock. Over time, additional capital, such as bioproduct capacity, biofuel, and cellulose hydrolysis, can be added to this emerging integrated PHA biorefinery [132,133]. In addition, biomass residues and fresh biomass could be converted to biofuel via thermochemical processes. According to the literature, the greenhouse gas emissions from switchgrass-based cellulosic C_2_H_5_OH is 94% less than that of gasoline [84]. Hence, PHAs are value-added coproducts obtained from the large scale production of biofuels and bioenergy from plants. Therefore, the synthesis of these biopolymers from biomass crops can considerably improve the economics of biomass biorefineries to produce liquid biofuels and bioenergy [131,134,135].

## 6. Conclusions and Future Directions

In conclusion, it has become clear that PHA-based biofuels are creating industrial value in energy, and the development of PHAs can address three basic issues:

1. Petroleum shortage for plastic materials.
2. Reduced CO2 emissions.
3. Environmental protection.

As a source of biofuels, PHAs are very promising biopolyesters since they do not need to be of high purity. Thus, PHAs can be obtained from crops, activated sludge, or nutritious wastewater making it cost-effective while also addressing the food vs. fuel and fuel vs. land controversies. Hence, PHAs are novel substrates for biofuel production where recent developments in PHAs synthesis concerning open and continuous mixed cultures will produce cost-effective PHAs for the biofuel applications. After being used as bioplastics, PHAs can undergo methyl esterification to biofuels, which further extends their application value. However, much work is still needed to make PHAs cost-effective so that PHAs-based biofuels can be used as an alternative to existing biofuels such as biodiesel, ethanol, methane gas, and hydrogen.

In the future, the production costs of PHAs should be taken into consideration to enhance their commercialization. The production of PHAs in activated sludge or wastewater should be improved. So far, P3HB is mostly used in the production of HAME biofuels, thus, more monomers should be explored and tested for esterification. It would be worthwhile to develop HAME into biolubricants or fuel additives for the diversification of the biofuel or fuel additive industry. In addition, upcoming research should also focus to improve the quality of HAME biofuels.

## Figures and Tables

**Table 1:** Comparison of polyhydroxyalkanoates (PHA) and polylactic acid (PLA).

Properties	PHA	PLA
Structure	Over 150 monomer units	D- and L-lactic acids (LA) monomer.
Synthesis method	Biosynthesized as intracellular polyester	Biological synthesis of LA and Chemical synthesis of PLA
Cost	Twice than PLA	Comparable with conventional plastics.
Material properties	Brittle (could be controlled by structural modifications)	Could be varied by adjusting D- and L- ratios.
Technology advancement	Over 10 companies Producing PHA up to 2000 ton/year by fermentation of microbes.	NatureWorks, WeforYou, Evonik, and Total-Corbion are largest PLA producers.
Applications	Almost all areas of conventional plastic industry.	Packaging, medical implants, printing and coatings.

**Table 2:** Bacterial strains for pilot and large-scale production of PHAs.

Strain	Carbon Source	DNA Manipulation	Final PHA (%)	Manufacturer
*Ralstonia eutropha*	Glucose	No	>80%	Tianjin North. Food, China
*Alcaligenes latus*	Glucose or sucrose	No	>75%	Chemie Linz, btF, Austria, Biomer, Germany
*E. coli*	Glucose	*P3HBCAB* + *vgb*	>80%	Jiang Su Nan Tian, China
*R. eutropha*	Glucose + propionate	No	>75%	ICI, UK, Zhejiang Tian An, China
*R. eutropha* *E. coli*	Glucose + 1,4-BD	No *P3HBCAB*	>75%	Metabolix, USA Escherichia coli P3HBCAB Tianjin Green Biosci. China
*R. eutropha*	Fatty acids	*PhaC_Ac_*	>80%	P&G, Kaneka, Japan
*Aeromonas hydrophila*	Lauric acid	No	<50%	P&G, Jiangmen Biotech Ctr, China
*A. hydrophila*	Lauric acid	*P3HBCAB* + *vgb*	>50%	Shandong Lukang,
*Pseudomonas putida,* *P. oleovorans*	Fatty acids	No	>60%	ETH, Switzerland
*Bacillus* spp.	Sucrose	No	>50%	Biocycles, Brazil
*R. eutropha +* recombinant *E. coli*	Glucose	No	>80%	Tianjin North. Food, China, and Lantian Group China
*Burkholderia* sp.	Sucrose	No	>70%	Industrial Usina da Pedra-Acucare Alcool Brazil
*A. latus*	Sucrose	No	>90%	Chemie Linz, Austria
*R. eutropha*	Glucose	No	>80%	NingBo TianAn, China
*A. hydrophila*	Glucose + Lauric acid	No	50%	Guangdong Jiangmen Center for Biotech Development, China, Procter & Gamble, USA

**Table 3:** Microbial production of PHAs homopolymers.

Sr No.	Homopolymer	Strain or Plasmid	Involved Genes/Genes Sequence	Carbon Source	Ref.
1	P3HB 3H4PE ^a^	*Burkholderia* sp.	*phaC*	Gluconate and sucrose	[59]
2	P3HB PHO ^b^ PHD ^c^	*Pseudomonas nitroreducens* AS 1.2343		Hexanoate and octanoate, butyrate, decanoate, lauric acid and tetradecanoic acid	[60]
3	P3HB	*Pseudomonas* sp. 61-3	*P3HB*	Glucose	[61]
4	P3HB	*R. eutropha* H16		Propionic acid	[62]
5	PHHp ^d^	*P. putida* KTOY06	*fadBA*	Heptanoate	[63]
6	PHV	*hydrophila* 4AK4	*vgb* and *fadD*	undecanoic acid	[64]
7	P3HB	Recombinant *E. coli*	*Sau3A* I	Glucose	[65]
8	P3HB P4HB PHV	*Bacillus cereus*	*16S rRNA*	Fructose, sucrose, and gluconate	[66]
9	P3HB	*C. necator* and *Burkholderia sacchari*		Glycerol and Glucose	[67]
10	P3HB	Recombinant *E. coli*	*phaCAB*	Cheese whey	[68]
11	P3HB	*R. eutropha* H16 and its recombinant strain	*phaC_Ac_*	Soybean oil	[69]
12	P3HB	*C. necator* strain A-04		Refined sugarcane, Brown sugarcane, Coconut palm sugar, rock sugar, toddy palm sugar and	[70]
13	P3HB PHV PHO PHDD ^e^	*Wautersia eutropha*		Canola oil	[71]
14	P3HB	*Pseudomonas hydrogenovora*		Lactose, glucose, and galactose	[72]
15	P3HB	*Bacillus firmus* NII 0830		Pineapple Crude glycerol	[73]
16	P3HB	*Bacillus* sp. SV13		and sugarcane	[74]
17	P3HB	*C. necator* DSM 545	*PhaA*	Soy cake and molasses.	[75]
18	P3HB	*C. necator* DSM 545	*PhaA*	Waste glycerol	[76]
19	P3HB	*W. eutropha*		Wheat based bio refinery	[77]
20	P3HB	*E. coli* DH5 α and KSYH(DE3)	*bktB-phaB-phaC* under *trc* promotor	Tryptone	[78]
21	PHV	*Bacillus* strain PJC48	*16S rDNA*	Glucose	[79]
22	PHDD	*P. putida* KT2440	*phaC1_Pp_*	Sodium dodecanoate	[80]
23	PHO	*P. putida* ATCC47054		Glycerol and sodium octanoate	[81]
24	P3HB	*Burkholderia* sp. AIU M5M02	*16S rRNA*	Mannitol	[82]
25	P3HB	*Burkholderia thailandensis*	*rhlA, rhlB* and *rlh*	Cooking oil	[83]

^a^ 3-hydroxy-4-pentenoic acid, ^b^ polyhydroxyoctanoate, ^c^ polyhydroxydecanoate, ^d^ polyhydroxyheptanoate ^e^ 3-hydroxydodecanoate.

**Table 4:** Microbial production of PHAs copolymers.

Sr No.	Copolymer	Strain or Plasmid	Involved Genes/Genes Sequence	Carbon Source	Ref.
1	P3HB-*co*-HA	*Pseudomonas* sp. 61-3	*phaC1*	Gluconate alkanoates	[89]
2	P3HB-*co*-HA	*P.* sp. 61-3		Glucose	[61]
3	P3HB-*co*-HV	Recombinant *E. coli*	*phaA*	Glucose propionate	[90]
4	P3HB-*co*-HHx, P3HB-*co*-HV-*co*-HHp	Recombinant *R. eutropha P3HB*–4	*P3HB* *phaC_Ac_*	Hexanoate and octanoate, pentanoate and nonanoate	[91]
5	P3HB-*co*-P4HB	*R. eutropha* H16		*n*-alkanoic acids	[62]
6	P3HB-*co*-HA	*R. eutropha* P3HB^-^4	*ph*aC1*_Ps_*	Fructose	[92]
7	P3HB-*co*-HHx	*A. hydrophila*		lauric acid, and oleic acid	[93]
8	P3HB-*co*-HHx	*A. hydrophila*	*phaC* coexpressed with *phaP phaJ*	Dodecanoate	[94]
9	P3HB-*co*-P4HB	*R. eutropha* ATCC 17699		Fructose and γ-butyrolactone	[95]
10	P3HB-*co*-P4HB	*necator*		Spent palm oil	[96]
11	P3HB-*co*-HHx	*R. eutropha* H16 and its recombinant strain	*phaCAc*	Soybean oil	[69]
12	P3HB-*co*-4HB	*C. necator* strain A-04		sugarcane, rock sugar, toddy palm and coconut palm sugar	[70]
13	P3HB-*co*-P3HV	*P. hydrogenovora*		Lactose, glucose and galactose	[72]
14	P3HB-*co*-P3HV-*co*-P3HHx	Recombinant *A. hydrophila* 4AK4	*phaA* and *phaB*	Dodecanoic acid and propionic acid	[97]
15	P3HB-*co*-P4HB	*Comamonas acidovorans*	*phaA*	Glucose and 1,4-butanediol	[98]
16	P3HB-*co*-P3HV	*C. necator* H16	*phaA*	palm oil and palm olein	[99]
17	P3HB-*co*-P3HV-*co*-P3HHx	Recombinant *C. necator*	*PhaC_Ac_*	Palm kernel oil	[35]
18	P(3HP-co-4HB) ^a^	Recombinant *E. coli*	*OrfZ, pcs, dhaT, aldD,* and *phaC1*	Glycerol	[100]
19	P3HB-*co*-P3HV	*Bacillus* sp.	16S rDNA	Glucose, glycerol, sod. acetate	[101]
20	P3HB-*co*-P3HV	*Bacillus cereus* FA11	16S rRNA	Glucose	[102]
21	P3HB-co-P3HHx	Recombinant *C. necator* strain Re2160/pCB113	*phaJ*	palm kernel oil, soybean oil, corn oil, and coconut oil	[103]
22	P3HB-co-P3HA	Recombinant R. eutropha	*phaC1_Ps_*	Soybean oil, fructose	[104]
23	P3HB-*co*-P4HB	*Burkholderia contaminans*	*phaC_Bcon_*	sodium-4-hydroxybutyrate	[105]
24	P3HB-*co*-P4HB	*Cupriavidus malaysiensis* USMAA1020	*phaC*	1,4-butanediol and 1,6-hexanediol	[106]
25	P3HB-*co*-P3HHx	*R. eutropha* Re2133/pCB81	*phaC2*	sodium acetate, sodium butyrate, sodium lactate and sodium propionate	[107]
26	P3HB-*co*-P3HHx	*R. eutropha* H16	*phaC2*	Butyrate	[108]
27	P3HB-*co*-P3HV	*E. coli* YH090	*atoAD* overexpressed with *bktB, phaB,* and *phaC*	propionate	[109]
28	P3HB-*co*-P3HHx	*R. eutropha* Re2133	*phaJ* and *phaC2*	coffee waste oil	[110]
29	P3HB-*co*-P3HV	*R. eutropha* 5119	*hmfH*	Glucose	[111]
30	P3HB-*co*-P3HHx	*R. eutropha* H16	*phaB1* and *phaB2*	Glucose, fructose and glycerol	[112]

^a^ poly(3-hydroxypropionate-co-4-hydroxybutyrate)

**Table 5:** Types of biofuels for energy.

Liquid	Solid	Gaseous
Biodiesel, Methanol, Ethanol, biobutanol gasohol, biogasoline, Hydrotreated Vegetable Oil (HVO), hydroxyalkanoates methyl ester (HAME) or hydroxybutyrate methyl ester (HBME)	Wood fuel, sawdust, charcoal and bagasse, dried animal dung	Methane (CH_4_), Butane (C_4_H_10_) and Hydrogen (H_2_) gas

## Data Availability

Not applicable.
